# Diagnostic Value of Single LH and LH/FSH Ratio at 60-minute after GnRHa Stimulation Test for Central Precocious Puberty

**DOI:** 10.1007/s12098-024-05137-7

**Published:** 2024-05-13

**Authors:** Qingling Wang, Dan Wu, Qian Zeng, Chuanwei Ban, Ling Wang, Xin Lv

**Affiliations:** 1 Clinical Laboratory, Childrenʼs Hospital Affiliated to Shandong University, 23976 Jing-Shi Road, Jinan, 250022 Shandong Province People’s Republic of China; 2 Clinical Laboratory, Jinan Childrenʼs Hospital, Jinan, China

**Keywords:** Central precocious puberty, Gonadotropin-releasing hormone analogs stimulation test, Diagnostic value, Luteinizing hormone, Follicle stimulating hormone

## Abstract

**Objectives:**

To evaluate the diagnostic value of luteinizing hormone (LH) and LH/follicle stimulating hormone (FSH) ratio at 60 min after gonadotropin-releasing hormone analogs (GnRHa) stimulation test for central precocious puberty (CPP) in girls.

**Methods:**

Two hundred and fifty-seven girls, aged 3 to 7.5 y, suspected of precocious puberty at authors’ hospital from April 2020 through November 2023 were enrolled in the study. The blood was taken at 0, 30, 60 min after GnRHa stimulation test, and LH and LH/FSH were detected by chemiluminescence assay. The diagnostic efficacy was analysed by Mann–Whitney U test, spearman’s correlation analysis and receiver operating characteristic (ROC) analysis. The proportion of obesity was analysed by Chi-square test.

**Results:**

LH and LH/FSH at different times were statistically significantly different (*P* <0.05) between the CPP and non-CPP groups. Spearman’s correlation analysis showed that the level of LH and LH/FSH at 60 min had the strongest consistency with the peak of LH (r = 0.9988, *P* <0.001) and LH/FSH (r = 0.9981, *P* <0.001). ROC curve analysis showed that the area under the ROC curves at 60 min of LH and LH/FSH were 0.975 and 0.997 with a cut-off value of 5.70 IU/L and 0.609, respectively.

**Conclusions:**

The peak of LH and LH/FSH in the diagnosis of CPP can be determined by LH and LH/FSH at 60 min after the triptorelin acetate is injected. This will reduce the number of blood draws required compared with the traditional stimulation test, which is more effective and acceptable for children.

## Introduction

Central precocious puberty (CPP) refers to hypothalamic-pituitary–gonadal axis (HPGA) initiation in advance because of the various factors, resulting in a higher level of gonadotropin-releasing hormone (GnRH), luteinizing hormone (LH) and follicle stimulating hormone (FSH), influencing on the ovary or testis, finally resulting in increased level of sex hormone, advanced clinical secondary characteristics and bone age [1].The incidence of precocious puberty in China was 11.47% in girls and 3.26% in boys [2]. A study showed that the incidence of CPP is about 1/5000–1/10000 in girls, which is about 5–10 times that of boys [3]. The incidence and prevalence of CPP in different races is different, but most of them showed an early onset age of secondary sexual characters. For example, female breast development is about 3 mo earlier every 10 y [4]. Due to the premature sexual development in CPP, the accelerated skeletal growth can lead to impaired growth potential of the children [5], and can cause psychological problems and social behavior abnormalities.

Expert consensus on the diagnosis and treatment of central precocious puberty in China (2022) proposed that CPP diagnosis is made at an early age according to the sexual characteristics (before 7.5 y for girls and before 9.0 y for boys), increased volume of gonads and advanced bone age, and higher growth speed than that of healthy children of the same age [6]. The gonadotropin-releasing hormone (GnRH) stimulation test is considered as the gold standard for the diagnosis of CPP in children with early symptoms of puberty [7].

The gonadotropin-releasing hormone analogs (GnRHa) stimulation test is considered to be more accurate than measuring basal LH levels between 8 to 9 am. It serves as a crucial tool in identifying CPP and peripheral precocious puberty. However, it is important to note that there are variations in the diagnostic criteria among different countries and ethnic groups. When GnRHa is used as the stimulating test drug, it is recommended to develop its own dosage criteria and diagnostic standard [6, 8]. In the past, GnRHa stimulation test usually required blood collections at 0, 30, 60, 90, 120 min to detect the concentration of LH and FSH, and the patients had to wait for a long time. In addition, it caused great physical and psychological pressure on the patients.

This study aimed to investigate the diagnostic efficacy of LH and LH/FSH ratio at 60 min after GnRHa stimulation test for CPP in girls in order to find a more economic, effective and acceptable diagnostic method for children.

## Material and Methods

Two hundred and sixty-six girls, aged 3 to 7.5 y, suspected of precocious puberty at authors’ hospital from April 2020 to November 2023, were selected and underwent GnRHa stimulation test. Eight cases were excluded due to organic diseases and 1 was excluded for some other disease and thus, 257 girls were enrolled in the study.

Inclusion standards: (1) early onset of sexual signs; girls having breast development before age 7.5 y, (2) longer uterus length (≥1.83 cm) and enlarged ovarian volume (≥1 mL) [9], and multiple ≥4 mm diameter follicles in the ovary, (3) hypothalamic and pituitary magnetic resonance was tested, and (4) underwent GnRHa stimulation test.

Exclusion standards: (1) patients with organic disease that could affect the function of HPGA, such as, pituitary tumor and ovarian cyst, (2) patients with other diseases.

The basic information, hematology indicators, and clinical characteristics of the study subjects were retrospectively collected and is summarized in Table 1.

**Table 1:** Clinical and laboratory characteristics of the study population

**Characteristics**	**Study population (n = 257)**
Age (years)	7.00 (6.67–7.33)
Height (m)	1.28 (1.24–1.33)
Height-SDS	1.40 (0.65–2.29)
Weight (kg)	26.70 (23.60–31.10)
Weight-SDS	1.56 (0.54–2.69)
BMI (kg/m^2^)	16.32 (15.13–18.27)
BMI-SDS	0.81 (0.08–2.01)
BA (years)	9.0 (8.08–9.50)
BA-CA (years)	2.00 (1.19–2.58)
LH (IU/L)	0.12 (0.07–0.44)
LH-Peak (IU/L)	5.23 (2.69–9.75)
FSH (IU/L)	2.40 (1.59–3.94)
FSH-Peak (IU/L)	9.88 (11.24–15.17)
LH/FSH-Peak	0.44 (0.26–0.86)
Estradiol (pg/mL)	10.0 (10.0–18.0)
Progesterone (ng/mL)	0.10 (0.10–0.20)
Prolactin (ng/mL)	9.00 (6.47–13.73)
fT3 (pmol/L)	5.79 (5.32–6.26)
fT4 (pmol/L)	13.24 (12.48–14.26)
TSH (µIU/mL)	2.03 (1.55–2.71)
Breast Tanner stage	B2 (75.1%), B3 (21.8%), B4 (3.1%)
Uterus length (cm)	2.2 (2.0–2.5)
Ovarian volume (mL)	2.08 (1.60–2.65)

Breast Tanner stage reported as percentage, other data reported as median (IQR), LH-Peak, FSH-Peak, LH/FSH-Peak refers to the peak level of LH, FSH, LH/FSH, respectively

*BA* Bone age, *BMI* Body mass index, *CA* Chronological age, *FSH* Follicle stimulating hormone, *fT3* Free triiodothyronine, *fT4* Free thyroxine, *LH* Luteinizing hormone, *TSH* Thyroid-stimulating hormone

The patient’s blood was collected between 8 to 9 am for basal hormone tests, including LH, FSH, estradiol, prolactin, progesterone, free triiodothyronine (fT3), free thyroxine (fT4), and thyroid-stimulating hormone (TSH), by the microparticle chemiluminescence assay (Abbott Ireland Diagnostic Division). Then triptorelin acetate (Changchun Jinsai Pharmaceutical Company) was subcutaneously injected, at a dosage of 2.5 µg/kg (maximum dosage = 100 µg). Blood samples were taken 30 and 60 min later and LH and FSH were determined. Patients with a peak of LH (≥5 IU/L) and a peak of LH/FSH (≥0.6) were defined as central precocious puberty, and assigned to CPP group. Those who did not meet these criteria were assigned to non-CPP group. Patients of the same age and sex, with a body mass index (BMI) higher than 95%, were diagnosed with obesity.

All statistical analyses were done using the statistical software SPSS version 25 software (SPSS; IBM). Mann–Whitney U test were used to compare the general data and hormones at different time in the two groups and the results are shown by median and interquartile range. Spearman’s correlation analysis was applied to detect the correlation of LH and LH/FSH in different time. Receiver operating characteristic (ROC) analysis was used to evaluate the diagnostic utility of basal LH, FSH and LH/FSH and their values 0, 30, 60 min after the triptorelin acetate was injected. Youden’s index was calculated by sensitivity plus specificity minus one; the higher index indicates better diagnostic efficacy. The percentage of obesity in the two groups were compared by Chi-square test. *P* <0.05 was considered as statistically significant different.

## Results

A peak of LH (≥5 IU/L) with a peak of LH/FSH (≥0.6) were defined as central precocious puberty. One hundred and one girls were enrolled in the CPP group and another 156 girls were enrolled in the non-CPP group. There was no significant difference in age, BMI and bone age between the two groups (*P* >0.05). Uterus length, ovarian volume and breast tanner stage were statistically significantly different between the two groups (*P* <0.05) (Table 2).

**Table 2:** Basic data and indicators of the hormone comparison of the two groups

	**CPP (n = 101)**	**Non-CPP (n = 156)**	***P* value**
Age (years)	7.08 (6.83–7.33)	7.00 (6.58–7.25)	0.056
Height (m)	1.28 (1.24–1.32)	1.28 (1.24–1.32)	0.798
Height-SDS	1.50 (0.59–2.33)	1.39 (0.67–2.18)	0.733
Weight (kg)	27.5 (23.5–31.65)	26.35 (23.7–29.63)	0.330
Weight-SDS	1.71 (0.48–2.95)	1.48 (0.58–2.32)	0.195
BMI (kg/m^2^)	16.59 (15.15–18.98)	16.29 (15.11–17.58)	0.240
BMI-SDS	0.97 (0.09–2.53)	0.79 (0.07–1.62)	0.245
BA (years)	9.10 (8.05–9.70)	8.90 (8.03–9.40)	0.171
BA-CA (years)	2.02 (1.22–2.60)	1.93 (1.13–2.52)	0.301
Breast Tanner stage	B2 (61.4%), B3 (32.7%), B4 (5.9%)	B2 (84.0%), B3 (14.7%), B4 (1.3%)	0.000
Uterus length (cm)	2.4 (2.1–2.6)	2.1 (1.9–2.3)	0.000
Ovarian volume (mL)	2.21 (1.70–2.83)	1.97 (1.56–2.60)	0.041
LH (IU/L)	0.44 (0.18–0.83)	0.08 (0.04–0.14)	0.000
LH-30 (IU/L)	11.79 (7.05–20.00)	2.81 (1.89–4.01)	0.000
LH-60 (IU/L)	12.60 (7.98–20.84)	3.02 (2.11–4.63)	0.000
LH-Peak (IU/L)	13.30 (7.98–21.21)	3.10 (2.13–4.68)	0.000
FSH (IU/L)	3.15 (1.94–4.45)	2.10 (1.48–3.33)	0.000
FSH-30 (IU/L)	9.76 (7.58–13.34)	8.82 (6.93–10.80)	0.004
FSH-60 (IU/L)	13.16 (9.60–17.17)	11.45 (8.94–14.14)	0.003
FSH-Peak (IU/L)	13.16 (9.60–17.17)	11.45 (8.94–14.14)	0.003
LH/FSH	0.04 (0.03–0.06)	0.13 (0.08–0.22)	0.000
LH/FSH-30	1.14 (0.86–1.58)	0.33 (0.23–0.47)	0.000
LH/FSH-60	0.97 (0.74–1.30)	0.27 (0.20–0.38)	0.000
LH/FSH-Peak	0.98 (0.77–1.36)	0.28 (0.20–0.39)	0.000
Estradiol (pg/mL)	13.0 (10.0–27.0)	10.0 (10.0–14.0)	0.000
Prolactin (ng/mL)	9.28 (6.49–14.95)	8.77 (6.48–12.62)	0.314
Progesterone (ng/mL)	0.1 (0.1–0.2)	0.1 (0.1–0.2)	0.917
fT3 (pmol/L)	5.83 (5.35–6.43)	5.77 (5.30–6.18)	0.193
fT4 (pmol/L)	13.03 (12.44–13.86)	13.50 (12.60–14.40)	0.033
TSH (µIU/mL)	2.13 (1.61–2.73)	1.90 (1.50–2.67)	0.269

LH-30, FSH-30, LH/FSH-30 refer to the results at 30 min; LH-60, FSH-60, LH/FSH-60 refer to the results at 60 min

*BA* Bone age, *BMI* Body mass index, *CA* Chronological age, *CPP* Central precocious puberty, *FSH* Follicle stimulating hormone, *fT3* Free triiodothyronine, *fT4* Free thyroxine, *LH* Luteinizing hormone, *TSH* Thyroid-stimulating hormone

LH, FSH and LH/FSH at 0, 30, 60 min after the triptorelin acetate was injected were statistically significantly different between the two groups (*P* <0.05). Estradiol and fT4 were also statistically significantly different between the two groups (*P* <0.05). There was no significant difference in prolactin, progesterone, fT3 and TSH between the two groups (*P* >0.05), as shown in Table 2.

**Fig. 1:**
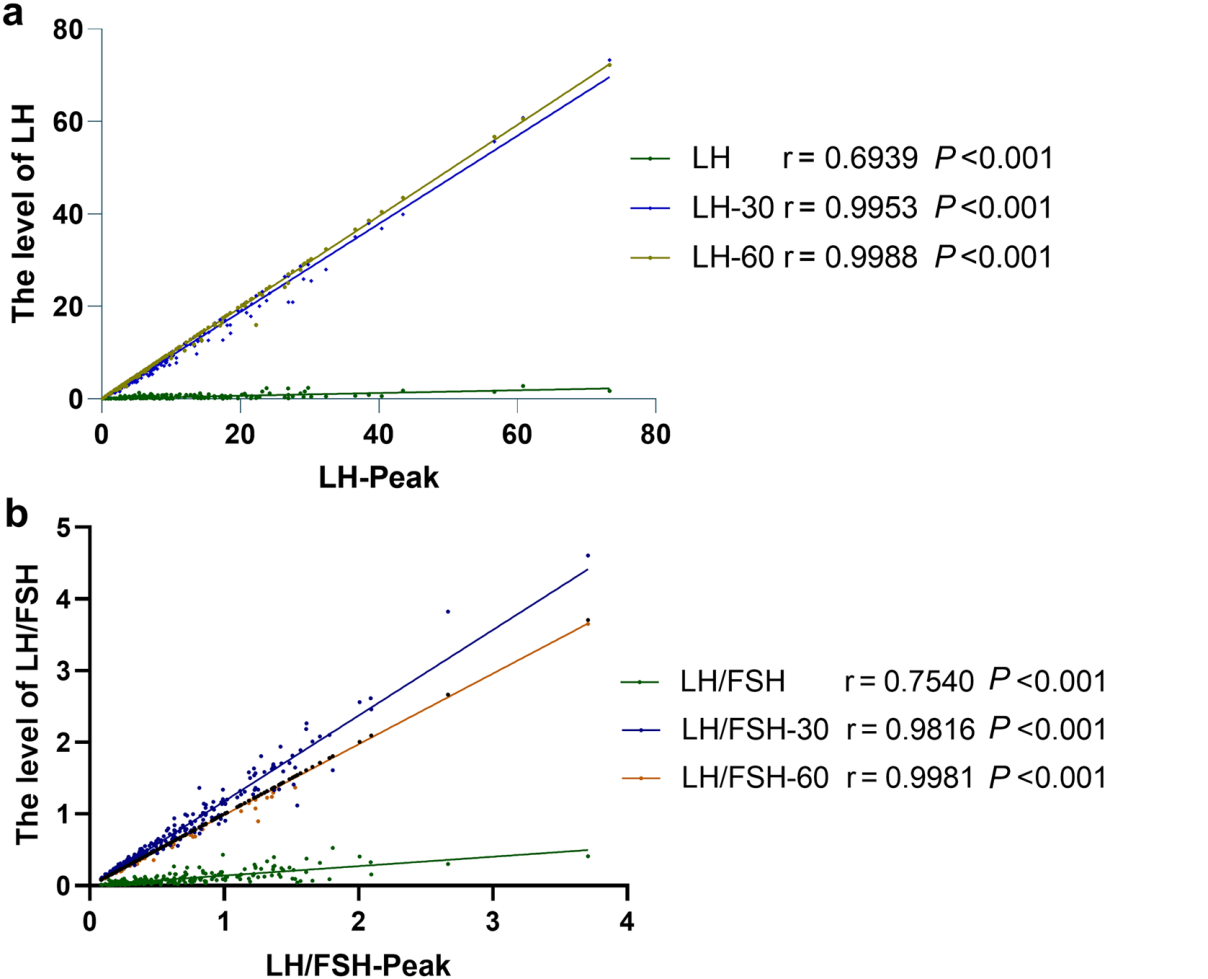
Spearman’s correlation analysis to detect the correlation of LH and LH/FSH at different times Correlation analysis of **a** LH peak and LH level, **b** LH/FSH peak and LH/FSH level. LH-30, LH/FSH-30 refer to the results at 30 min; LH-60, LH/FSH-60 refer to the results at 60 min. *FSH* Follicle stimulating hormone, *LH* Luteinizing hormone

Spearman’s correlation analysis showed positive correlations between LH peak and LH, LH-30, LH-60, and the strongest correlation for LH-60 (r = 0.9988, *P* <0.001). The LH/FSH correlation analysis also showed positive correlations between LH/FSH peak and LH/FSH, LH/FSH-30, LH/FSH-60, and the strongest correlation for LH/FSH-60 (r = 0.9981, *P* <0.001), as shown in Fig. 1.

**Fig. 2:**
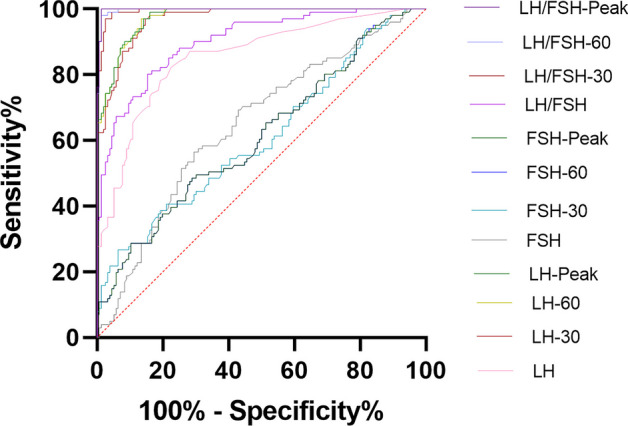
ROC curves of the diagnostic efficiency of LH, FSH, and LH/FSH at different time points. LH-30, FSH-30, LH/FSH-30 refer to the results at 30 min; LH-60, FSH-60, LH/FSH-60 refer to the results at 60 min. *FSH* Follicle stimulating hormone, *LH* Luteinizing hormone

ROC curves were used to evaluate the diagnostic utility including sensitivity and specificity of LH, FSH, LH/FSH at 0, 30, 60 min after triptorelin acetate was injected. Meanwhile, ROC curves were also used to generate the optimal cut-off value of hormone for the diagnosis of central precocious puberty. The highest area under the ROC curve (AUC) was LH/FSH peak (0.998) with a cut-off value of 0.605 (sensitivity 100%, specificity 98.7%). The AUC of LH/FSH level (0.997) at 60 min after triptorelin acetate was injected was almost equal to the peak level of LH/FSH (0.998). The Youden’s index of LH peak (0.829) was lower than LH at 60 min (0.836) after the triptorelin acetate was injected. The optimal cut-off value of LH peak for the diagnosis of central precocious puberty was 5.675 IU/L (sensitivity 97.0%, specificity 85.9%). The ROC curves are shown in Fig. 2 and Table 3.

**Table 3:** The optimal cut-off values of hormones and their diagnostic utility for central precocious puberty

	**AUC**	**95% CI**	***P* value**	**Cut-off**	**Sensitivity**	**Specificity**	**Youden’s index**
LH	0.855	0.807 ~ 0.903	<0.001	0.145	0.822	0.776	0.597
LH-30	0.969	0.952 ~ 0.986	<0.001	5.185	0.970	0.853	0.823
LH-60	0.975	0.960 ~ 0.989	<0.001	5.700	0.970	0.865	0.836
LH-Peak	0.975	0.961 ~ 0.989	<0.001	5.675	0.970	0.859	0.829
FSH	0.647	0.579 ~ 0.716	<0.001	2.815	0.564	0.705	0.270
FSH-30	0.608	0.536 ~ 0.679	0.0036	13.280	0.267	0.936	0.203
FSH-60	0.611	0.540 ~ 0.682	0.0027	13.660	0.485	0.712	0.197
FSH-Peak	0.611	0.540 ~ 0.682	0.0027	13.660	0.485	0.712	0.197
LH/FSH	0.903	0.866 ~ 0.940	<0.001	0.074	0.802	0.846	0.648
LH/FSH-30	0.995	0.989 ~ 1.000	<0.001	0.642	0.990	0.955	0.945
LH/FSH-60	0.997	0.993 ~ 1.000	<0.001	0.609	0.980	0.987	0.967
LH/FSH-Peak	0.998	0.995 ~ 1.000	<0.001	0.605	1.000	0.987	0.987

LH-30, FSH-30, LH/FSH-30 refer to the results at 30 min; LH-60, FSH-60, LH/FSH-60 refer to the results at 60 min

*AUC* Area under the curve, *FSH* Follicle stimulating hormone, *LH* Luteinizing hormone

There was no statistically significant difference in BMI (*P* >0.05) and BMI-SDS (*P* >0.05) between the two groups. However, the percentage of obesity in the CPP group (34.65%) and non-CPP group (19.23%) was statistically significantly different (χ^2^ = 7.718, *P* <0.05).

## Discussion

CPP refers to hypothalamic-pituitary–gonadal axis initiation in advance, elevated level of gonadotropin hormone, LH and FSH, and presence of clinical manifestations of secondary sexual development [1]. Many countries around the world show onset of precocious puberty at an early age [4, 10, 11]. The expert consensus in China has modified the age of diagnosis of CPP from age 8 y to 7.5 y in girls [6], and based on this modification, this study selected girls under 7.5 y.

Diagnosis of CPP is challenging and requires multidisciplinary approaches; blood indicators were essential. Elevated level of blood basal LH is an important marker of initiation the HPGA. The value of random LH was emphasized in international consensus on GnRHa application published in 2019 for the diagnosis of CPP, suggesting that puberty initiation should be indicated by serum LH ≥0.83 IU/L (detected by chemiluminescence) in children with precocious puberty [12]. In the present study, patients with basal LH ≥0.795 IU/L (specificity 100%) were diagnosed as CPP in combination with the presence of clinical manifestations of secondary sexual development. In addition, basal level of LH could be affected by day and night rhythm and different Tanner stage; thus, a single LH elevation is not sufficient for diagnosis [13, 14].

GnRH-stimulation test is considered as the gold standard for the diagnosis of CPP [7]. The expert consensus in China expressed that the peak level of LH ≥5 IU/L, with the peak of LH/FSH ≥0.6 can be diagnosed as CPP [6], but there exists another cut-off value of the LH peak from 3.3–10.0 IU/L [15–17]. In the present study, a cut-off value of the LH peak was 5.675 IU/L (sensitivity 97.0%, specificity 85.9%), higher than the standard used in the present study but lower than the recommendations in other countries. The main purpose of this study was not to find a cut-off value of LH peak, but to explore whether the GnRHa stimulation test could be simplified and to find a more cost-effective method for CPP diagnosis.

Freire et al. conducted a GnRHa stimulation test to measure LH level at 3 h post triptorelin injection [8], while Cipolla et al. measured LH at 2 h after gonadotropin injection [17]. Poomthavorn et al. found that the peak LH level was tested at 1 h post triptorelin injection [18]. Previous studies showed that blood sample should be taken at 1 h, 2 h, and 3 h to detect the level of LH [8, 19]. If necessary, the level of estradiol should be combined for the diagnosis 24 h later. However, this approach had the disadvantage of making patients wait for an extended period of time, subjecting them to both physical and psychological pressure. Previous few studies have addressed whether GnRHa stimulation test can reduce the number of times blood is drawn and shorten the time of diagnosis. A retrospective study showed that blood samples can be taken only at 0, 30, and 60 min after a GnRHa stimulation test; blood collection at 90 and 120 min can be abolished [20].

The results in the present study showed that LH and LH/FSH at 0, 30 and 60 min had positive correlations with the peak of LH and LH/FSH, but the results at 60 min had the highest consistency with the peak of LH (r = 0.9988, *P* <0.001) and LH/FSH (r = 0.9981, *P* <0.001). Youden’s index of LH at 60 min (0.836) was higher than the index of LH peak (0.829) and LH at 30 min (0.823). The AUC of LH at 60 min was 0.975 (95% CI, 0.960 ~ 0.989), equal to the AUC (0.975, 95% CI, 0.961 ~ 0.989) of LH peak. Post researches about CPP diagnosis also focus on the value of LH peak, and ignored the importance of LH/FSH [17]. In the present study, LH/FSH showed more efficiency in the diagnosis of CPP. The AUC (0.998, 95% CI, 0.995 ~ 1.000) of LH/FSH peak was higher than the AUC (0.975, 95% CI, 0.961 ~ 0.989) of LH peak. The AUC (0.997, 95% CI, 0.993 ~ 1.000) of LH/FSH at 60 min is almost equal to the AUC (0.998, 95% CI, 0.995 ~ 1.000) of LH/FSH peak. It means the results of LH peak and LH/FSH peak can be replaced by the results at 60 min.

Although LH and LH/FSH at 30 min also had a high diagnostic efficiency, but lower than the same at 60 min because most of the peaks are present at 60 min. The cut-off values of LH and LH/FSH at 60 min were 5.70 IU/L (sensitivity 97.0%, specificity 86.5%) and 0.609 (sensitivity 98.0%, specificity 98.7%), respectively. In addition to LH and LH/FSH, the level of fT4 in CPP group was lower than non-CPP group, while the level of estradiol in CPP group was higher than non-CPP group; the conclusion is consistent with a previous study [17].

In the present study, there was no statistically significant difference in age and bone age between the two groups. But the bone age in both the groups were advanced, suggesting that bone age is helpful in the diagnosis of precocious puberty, had a lower specificity to distinguish CPP and peripheral precocious puberty.

Although previous studies have reported a negative correlation between BMI and the peak of LH [21–25], the present study did not find any significant differences between BMI and the peak of LH, perhaps because of the small number of the present study; thus, the result analysis was affected. There was no statistically significant difference in BMI (*P* >0.05) between the two groups, but the proportion of obesity has significant differences (χ^2^ = 7.718, *P* <0.05) in the present study. The relationship between BMI and precocious puberty remains controversial [26, 27].

Above all, this study was a retrospective study with a small sample size and only females were enrolled, resulting in certain limitations of this study. Further prospective studies should be performed with expanded sample size and population to ensure the accuracy and richness of this study.

## Conclusions

Patients with basal level of LH ≥0.795 IU/L can be diagnosed as CPP combined with clinical manifestations of secondary sexual development. The optimal cut-off value of LH peak was 5.675 IU/L. The peak of LH and LH/FSH in the diagnosis of CPP can be determined instead by LH and LH/FSH at 60 min after the triptorelin acetate is injected; the diagnostic accuracy is not affected. In other words, the blood should be taken only at 0 and 60 min after the triptorelin acetate is injected. This will reduce the number of times blood is drawn compared with the traditional stimulation test, which is more effective and acceptable method for children.
